# Global prevalence and predictors of scabies among prisoners: systematic review and meta-analysis

**DOI:** 10.1186/s12889-024-19401-0

**Published:** 2024-07-15

**Authors:** Amare Mebrat Delie, Eyob Ketema Bogale, Tadele Fentabel Anagaw, Misganaw Guadie Tiruneh, Eneyew Talie Fenta, Ousman Adal, Natnael Kebede

**Affiliations:** 1 Department of Public Health, College of Medicine and Health Sciences, Injibara University, Injibara, Ethiopia; 2 Department of Health Promotion and Behavioral Science, School of Public Health, College of Medicine and Health Sciences, Bahir Dar University, Bahir Dar, Ethiopia; 3 Department of Health Systems and Policy, Institute of Public Health, College of Medicine and Health Sciences, University of Gondar, Gondar, Ethiopia; 4 Department of Emergency and Critical Care Nursing, College of Medicine and Health Sciences, Bahir Dar University, Bahir Dar, Ethiopia; 5 Department of Health Promotion, School of Public Health, College of Medicine Health Sciences, Wollo University, Dessie, Ethiopia

**Keywords:** Human scabies, Global, Prevalence, Prisoners, Systematic review and meta-analysis

## Abstract

**Introduction:**

Scabies is a widespread issue in prisons due to overcrowded living conditions and limited healthcare resources. A recent study published in the Journal of Infection and Public Health discovered that the prevalence of scabies varies greatly among prisoners in different regions and facilities. This review aimed to determine the global prevalence and predictors of scabies among prisoners by conducting a systematic review and meta-analysis.

**Methods:**

We followed the Preferred Reporting Items for Systematic Reviews and Meta-Analysis checklist to report the findings of our systematic review and meta-analysis. Relevant databases including PubMed, Cochrane Library, ScienceDirect, and other grey literature databases were used to search and retrieve articles. The study included both published and unpublished research written in English languages for studies reporting the prevalence of human scabies among prisoners. This review has been registered on PROSPERO. The heterogeneity of the data was evaluated using the I^2^ statistic. A meta-analysis was conducted using STATA 17 software, with a 95% confidence interval. The researchers also conducted publication bias and sensitivity analysis.

**Results:**

The review included 7 studies involving 1, 309,323 prisoners. All included studies involved cross-sectional study design. The prevalence of scabies among prisoners ranges from 0.72% in Italy to 41.01% in Cameroon. The global pooled prevalence of human scabies among prisoners was found to be 6.57% (95% CI; 2.16–19.94). According to subgroup analysis, the overall prevalence of scabies among African prisoners was 19.55% (95% CI; 9.44–40.45), while the prevalence among prisoners outside of Africa was 1.57% (95% CI; 0.77–3.19). The length of time spent in prison, sharing of clothing or beds, and hygiene practices were found to be factors that were significantly associated with the likelihood of prisoners developing human scabies.

**Conclusion:**

The overall prevalence of human scabies is high among prisoners worldwide. Prisoners who spent more time in prison shared clothing or beds, and had poor hygiene practices were more likely to develop human scabies. Thus, efforts should be made by policymakers and program administrators to decrease the prevalence of scabies in prisons. The protocol for this systematic review and meta-analysis was registered in the International Prospective Register of Systematic Reviews with registration number CRD42024516064.

**Supplementary Information:**

The online version contains supplementary material available at 10.1186/s12889-024-19401-0.

## Background

Sarcoptes scabiei, formerly known as *Acarus scabiei*, was originally classified under the genus *Acarus*. It was later reclassified under the genus *Sarcoptes*, which is part of the superfamily *Sarcoptoidea* and the family *Sarcoptidae* [1]. Different types of *S. scabiei* mites can infect various animals, including humans, dogs, rabbits, and red foxes [2]. Human scabies is an ectoparasitic infestation caused by the mite *Sarcoptes scabies var. hominis* which is an obligate parasite that completes its entire life cycle on humans. Female mites burrow into the skin and lay eggs, eventually triggering a host immune response that leads to intense itching and rash [3, 4]. These mites can survive for up to 24–36 h at temperatures of 21 °C and humidity levels of 40–80%, and can still infect others during this time [5]. These mites can remain contagious for up to a week at lower temperatures and higher humidity and can enter the skin when the temperature is above 20 °C [6]. Scabies are more likely to spread in environments where there is prolonged direct contact and overcrowding, such as prisons, military camps, and boarding schools [7, 8].

Different regions and countries have different standards for the amount of space allocated per person in prison cells, with Europe requiring 4 square meters per person, and Australia and New Zealand requiring 5.75 square meters per person. For single cells, the range of space recommendations is from 2.4 square meters in Korea to 16 square meters in Switzerland, while for multiple-occupancy cells, the range is from 1.25 square meters in Pakistan to 10 square meters in the Netherlands. Moreover, prison cell sizes in African countries vary, with Kenya having 3.7 square meters for double cells, Senegal with 3.55 square meters, Guinea with 2 square meters, Malawi with 2–4 square meters, Mauritius with 4.08 square meters, and South Africa with 5.5 square meters for single cells and 3.5 square meters for multiple cells [9].

The Global Prison Trends 2023 report by Non-Governmental Organization Penal Reform International reveals that the global prison population has reached a record high of 11.5 million, leading to overcrowding in approximately 120 countries [10]. Africa is particularly affected, with countries like the Republic of Congo experiencing overcrowding levels of over 600%. Other countries facing high occupancy rates include Haiti (401%), Uganda (374%), France (119%), the Philippines (375%), and Cambodia (350%) [10]. The number of women and girls in prison has also significantly increased, with a nearly 60% rise from 2000 to 2022, totaling over 740,000 individuals [10]. Studies have shown that the prison population, which is predominantly made up of young people and adults, is more likely to experience skin problems due to overcrowding and poor hygiene in prisons. Prisons are a potential reservoir for various skin diseases, particularly scabies. These may spread to the community through visitors, workers, or the released inmates [11, 12]. It can affect individuals from various socio-economic backgrounds, regardless of age, gender, or race [5]. Scabies can also be transmitted indirectly through objects like bedding, towels, and clothing [13]. Outbreaks frequently occur in institutions and enclosed communities like prisons, regardless of their income level [14]. These outbreaks can have significant health and economic consequences and are challenging to manage, especially in crowded settings [14].

Scabies symptoms typically appear 4–6 weeks after being infested, although sometimes there may be visible signs before symptoms manifest [15]. The main feature of scabies is generalized itching that is more intense during nighttime, which may lead to absenteeism from work, and sleep disturbance that affects the quality of life and causes stigma [16, 17]. A diagnosis of scabies can be confirmed, clinical, or suspected, but clinical or suspected diagnoses should only be made if other possible conditions are ruled out [18]. A confirmed diagnosis of scabies can be made by identifying the scabies mite, eggs, or fecal pellets through microscopic examination of skin samples, high-magnification devices, or dermoscopy [18, 19]. Moreover, clinical scabies can be diagnosed if at least one of the following criteria is present: scabies burrows, typical lesions on the male genitalia, typical lesions in a typical distribution, and two relevant history features [18]. Scabies are suspected if a person has typical lesions in a typical distribution along with one history feature, or if they have atypical lesions or distribution along with two history features. The history features include itching or a positive contact history [18]. Scabies lesions are typically found on the skin below the mid-upper arm and thigh in older children and adults, as well as in the groin, breast, and peri-umbilical areas. The hands, fingers, and wrists are common areas for lesions, while infants may have lesions on the trunk, scalp, palms, and soles [18].

In individuals with weakened immune systems, scabies can develop into a severe form known as crusted scabies, which is caused by an excessive infestation of the same mite responsible for scabies [20]. Scabies infestations typically involve 5 to 15 mites, but individuals with crusted scabies can have thousands to millions of mites [5], making them highly contagious and able to cause outbreaks. Crusted scabies can range from mild to severe [5, 21, 22] and can lead to bacterial infections, which can cause serious health issues such as glomerulonephritis, rheumatic heart disease, sepsis, and even death, due to the openings in the skin [23].

The primary recommended treatment for scabies is a topical medication, usually permethrin 5% cream or benzyl benzoate 25% lotion [7, 8, 24]. Ivermectin, which is taken orally, is recommended as a secondary option for treatment [25]. Scabies are a common infectious disease that can be easily treated with a scabicide such as 5% permethrin [8, 24]. It is crucial to also provide treatment for anyone who has come into contact with the infected patient and to properly clean clothing and furniture to prevent the spread of infection [26].

Scabies is a widespread neglected tropical disease, with approximately 450 million new cases each year worldwide [3, 27, 28]. While scabies is more common in developing countries [7, 29], outbreaks in developed countries also contribute significantly to the global burden of the disease [30]. The prevalence of scabies varies widely, with the highest prevalence recorded in Papua New Guinea (71%), Panama (32%), and Fiji (32%), according to a global systematic analysis of population-based surveys [27]. In developed countries, the prevalence of scabies was typically substantially lower, with very few estimates above 2–4% [27, 31, 32]. Moreover, a previous worldwide systematic review which was conducted in 2022 found that the occurrence of scabies varies, with rates as low as 0.18% in Uganda and as high as 76.9% in Indonesia [33]. Another systematic review and meta-analysis revealed that the pooled global prevalence of scabies was 14.0% [34]. It was also found that the prevalence of scabies among prisoners differs greatly among different countries. It ranges from 0.72% in Italy [35] to 41.1% in Cameroon [36]. Scabies was recently recognized by the WHO as an Neglected Tropical Disease and included as part of the WHO roadmap for Neglected Tropical Disease 2021–2030 [37]. Although there are effective treatments for scabies [38], preventing and controlling the spread of the infestation in the population is difficult due to frequent re-infestation through community and personal interactions [7]. Besides the World Health Organization (WHO), the International Alliance for the Control of Scabies (IACS) [18] and informal research groups like the Sarcoptic-World Molecular Network are dedicated to collaborating towards the worldwide eradication of scabies [39]. Scabies is often overlooked in health control programs and research, despite its high prevalence [40].

Previous studies on human scabies in prisoners have yielded inconsistent results worldwide. This systematic review and meta-analysis aimed to determine the overall prevalence of human scabies among prisoners globally, as it is a significant issue in many prisons. Understanding this prevalence can help inform strategies for controlling and reducing the burden of scabies in prison populations. Policymakers and program implementers need to understand the prevalence of scabies in these settings to implement effective strategies for prevention and control. This study is the first of its kind to systematically review and analyze the prevalence of scabies among prisoners globally.

## Methods and materials

### Search strategy

A search strategy was implemented using electronic databases (PubMed, Science Direct, Cochrane Library, Google Scholar, and Grey Literature) which were systematically searched online to retrieve related articles using keywords. A comprehensive database search was performed using the Boolean operators “OR”, “AND”, and keywords. The literature search technique was conducted by using the keywords (“prevalence” OR “magnitude’’ OR “burden” OR “epidemiology” OR “predictor” OR “determinants” OR “associated factors” OR “factors” OR “causes”) AND (“human scabies” OR “scabies” OR “*Sarcoptes scabiei”* OR “skin disease” OR “skin infection” OR “dermatosis” OR “ectoparasite”) AND (“penitentiary” OR “prison” OR “prisoner” OR “prisoners” OR “imprisonment” OR “jail” OR “criminals” OR “convicts” OR “confined area” OR “inmates” OR “detainees” OR “offenders” OR “incarcerated” OR “detention”). We searched all articles, whether they were published or unpublished, until March 17, 2024. Grey literature was searched in institutional repositories and ResearchGate. The search was conducted using search terms related to the prevalence of human scabies among prisoners. Searches were conducted from March 1, 2024, to March 17, 2024. All original articles concerning human scabies among prisoners were reviewed for eligible studies. We used the Preferred Reporting Items for Systematic Reviews and Meta-Analysis(PRISMA) checklist to present the results of our systematic review and meta-analysis [41]. This systematic review and meta-analysis were performed by following Preferred Reporting Items for Systematic Reviews and Meta-Analyses (PRISMA) guidelines [42]. The protocol for this systematic review and meta-analysis is registered in the International Prospective Register of Systematic Reviews (PROSPERO) and obtained a registration number CRD42024516064.

### Inclusion and exclusion criteria

All English-language, full-text, original research articles and doctoral dissertations with observational study design (cross-sectional, case-control, or cohort study design) conducted among prisoners with no limit to certain geographical areas with adults, children, or adolescents, that were published in peer-reviewed journals or filed as completed dissertations until March 17, 2024, were eligible for inclusion. In contrast, qualitative studies, surveys, editorials, reports, and studies in which their prevalence was computed among those patients who had skin lesions were excluded from this study.

### Data extraction

After searching in relevant databases, articles was imported into Endnote version 20 and duplicates were removed. After initial screening, three reviewers (AMD, EKB, and TFA) downloaded abstracts to assess them for inclusion. If reviewers disagreed about whether a search result was relevant to the study, it was included for retrieval. Additionally, the abstracts’ compliance with the inclusion criteria was evaluated. At this stage, articles considered irrelevant or out of the scope of the study were excluded and the full text of the remainder was downloaded for a detailed review. In this review, if the reviewers were unsure about including or excluding an article based on the abstract, another author would decide. However, there were no disagreements or uncertainties between reviewers in this particular review regarding the inclusion or exclusion of articles based on their abstracts.

### Data quality assessment

The Joanna Briggs Institute (JBI) critical appraisal checklist was used to assess the quality of the studies. Using this tool as a protocol, four reviewers (AMD, MGT, and ETF, ) then assessed the quality of potentially eligible articles using the Joana Briggs Institute (JBI) criteria. Papers are screened for inclusion based on title, abstract, and other relevant information and then undergo a thorough evaluation before being included in the final review. The average of those independent reviewers’ scores was used to determine whether the articles should be included. Discrepancies in quality assessment scores were resolved with a third reviewer (OA), whenever appropriate. Those studies with scores of 5 or more in JBI criteria were considered to have good quality and were included in the review [43]. The study’s researchers attempted to get in touch with the authors of the articles twice in case more information was required, such as when patient outcome statistics were lacking (Table 1).

**Table 1:** Methodological quality assessment of included studies using the JBI critical appraisal checklist

Study	Inclusion in the sample clearly defined	Study subjects and the setting described in detail	Exposure measured in a valid and reliable way	Objective, standard criteria for measurement of the condition?	Confounding factors identified	Strategies to deal with confounding factors stated	Outcomes measured in a valid and reliable way	Was appropriate statistical analysis used?	Total score(*n* = 8)
Kouotou, EA [44]	Yes	Yes	Yes	Yes	Yes	Yes	Yes	Yes	8
Bartosik, K [45]	Yes	Yes	Yes	Yes	No	No	Yes	Yes	6
Bogino, E [46]	Yes	Yes	Yes	Yes	Yes	Yes	Yes	Yes	8
RAHMATI, R [47]	Yes	Yes	Yes	Yes	Yes	Yes	Yes	Yes	8
Mannocci, A [35]	Yes	Yes	Yes	Yes	Yes	Yes	Yes	Yes	8
Harouna, M [48]	Yes	Yes	Yes	Yes	Yes	Yes	Yes	Yes	8
Kouotou, E [36]	Yes	Yes	Yes	Yes	Yes	Yes	No	Yes	7

### Data analysis

Information on the study characteristics (Name of author/s, publication year, study location, study design, sample size, prevalence, and significant factors associated with scabies) was extracted from each study using Microsoft Excel Version 2019 and the extracted data were exported to STATA version 17 software for analysis. Data were summarized by tables and forest plots. The standard error and 95% confidence interval for the prevalence of scabies patients were calculated for those studies in which estimates of standard error and 95% confidence interval for their proportion were not found in the full text of the article. The statistical heterogeneity was checked subjectively by using forest plots, and objectively by Cochrane Q-test and _I_^2^ statistics [49]. A meta-analysis based on a random effect model was applied to determine the pooled prevalence of human scabies infection in prison because significant and considerable heterogeneity exists between studies (I^2^ = 99.78%, Q = 390.33, *p* < 0.001). The presence of publication bias was checked by using a funnel plot and Egger’s and Begg’s statistical tests [50]. In this study, Egger’s and Begg’s tests at a 5% significant level were not significant for publication bias. However, the Doi plot revealed major asymmetry with an LFK index of 8.92, indicating the presence of publication bias. Subgroup analysis was also conducted based on the study setting.

## Result

**Fig. 1:**
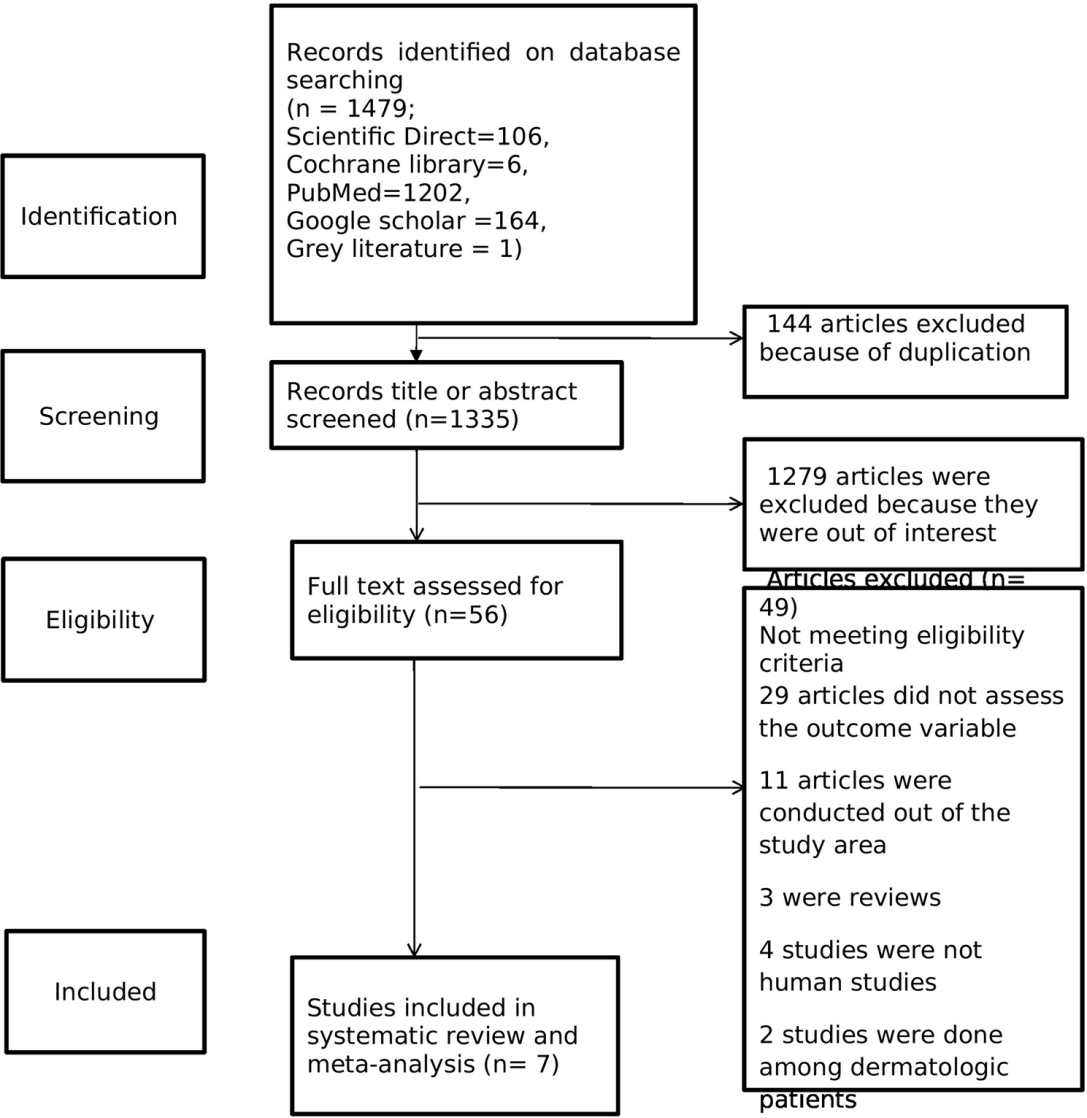
PRISMA flow diagram of study selection of global pooled prevalence of human scabies among prisoners,2014

A total of 1479 records were identified through our initial database search. After duplicate records were removed, 1355 records were reviewed by title and abstract. Fifty-six articles were included for full-text review. After applying inclusion and exclusion criteria, a total of seven studies were finally included in the review (Fig. 1). No additional studies were obtained after retrieving the references of the 7 included articles.

## Study characteristics

A meta-analysis was conducted on seven studies published between 2014 and 2023. These studies were carried out in various countries, including Cameroon [36], Poland [45], Ethiopia [46], Iran [47], Italy [35], Hungary [51], and Niger [48]. All of the studies were cross-sectional [35, 36, 45–48, 51]. The total sample size for this review was 1,309,323 with a maximum sample size of 1,302,481 in Poland [45], and a minimum sample of 217 in Cameroon [36]. The highest occurrence of scabies infestation was found in Cameroon at a prevalence rate of 41% [36], while the lowest occurrence was in Italy at a rate of 0.72% [35] (Table 2).

**Table 2:** Characteristics of the included studies in meta-analysis for the global prevalence of human scabies infestation, 2024

Serial number	Author	Publication Year	Study design	Study setting	Country	Number of scabies cases	Sample size	Prevalence in %
1.	Kouotou, EA [36]	2016	cs	Prison	Cameroon	89	217	41.01
2.	Bartosik, K [45]	2020	cs	Prison	Poland	28,943	1,302,481	2.24
3.	Bogino, E [46]	2023	cs	Prison	Ethiopia	37	416	8.85
4.	RAHMATI, R [47]	2007	cs	Prison	Iran	31	1404	2.21
5.	Mannocci, A [35]	2014	cs	Prison	Italy	19	2653	0.72
6.	Harouna, M [48]	2023	cs	Prison	Niger	43	352	12
7.	Kouotou, E [36]	2018	cs	Prison	Cameroon	242	755	32.05

Note that: cs: Cross-sectional study design

### Overall prevalence of scabies among prisoners

**Fig. 2:**
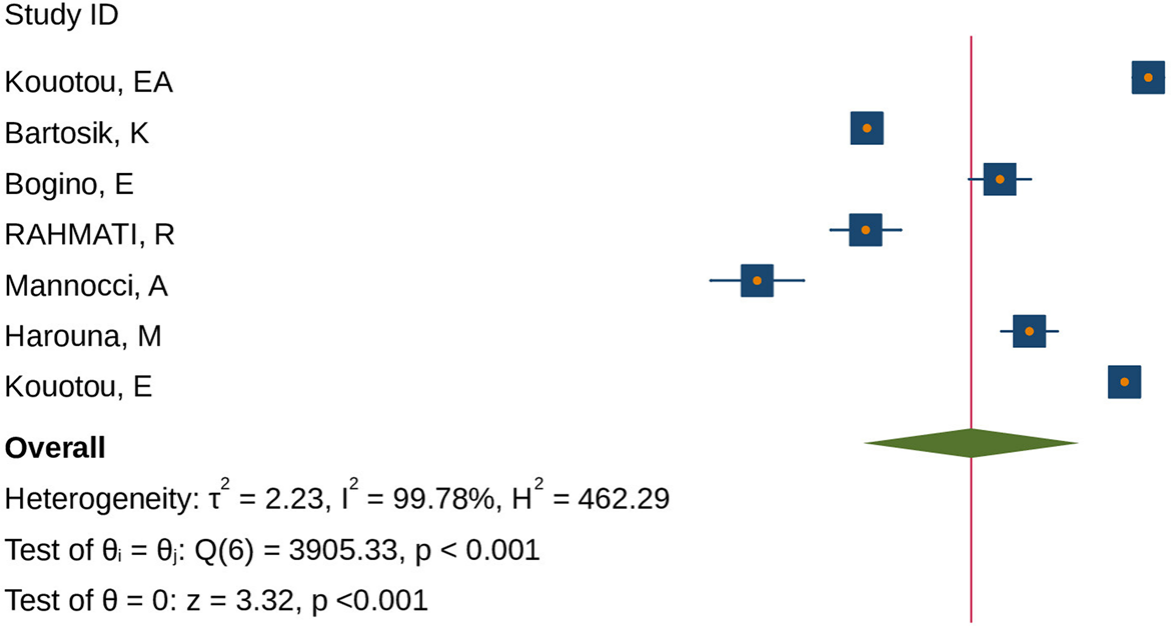
The pooled global prevalence of humab scabies among prisoners,2024

From the included studies in this systematic review, the lowest prevalence (0.72%) was found in a study conducted in Italy [35] whereas the highest prevalence of scabies (41.01%) was found in a study conducted in Cameroon [44]. In this review, the pooled prevalence of scabies among prisoners were found to be 6.57% (95% CI; 2.16–19.94) (Fig. 2).

### Subgroup analysis based on study area

**Fig. 3:**
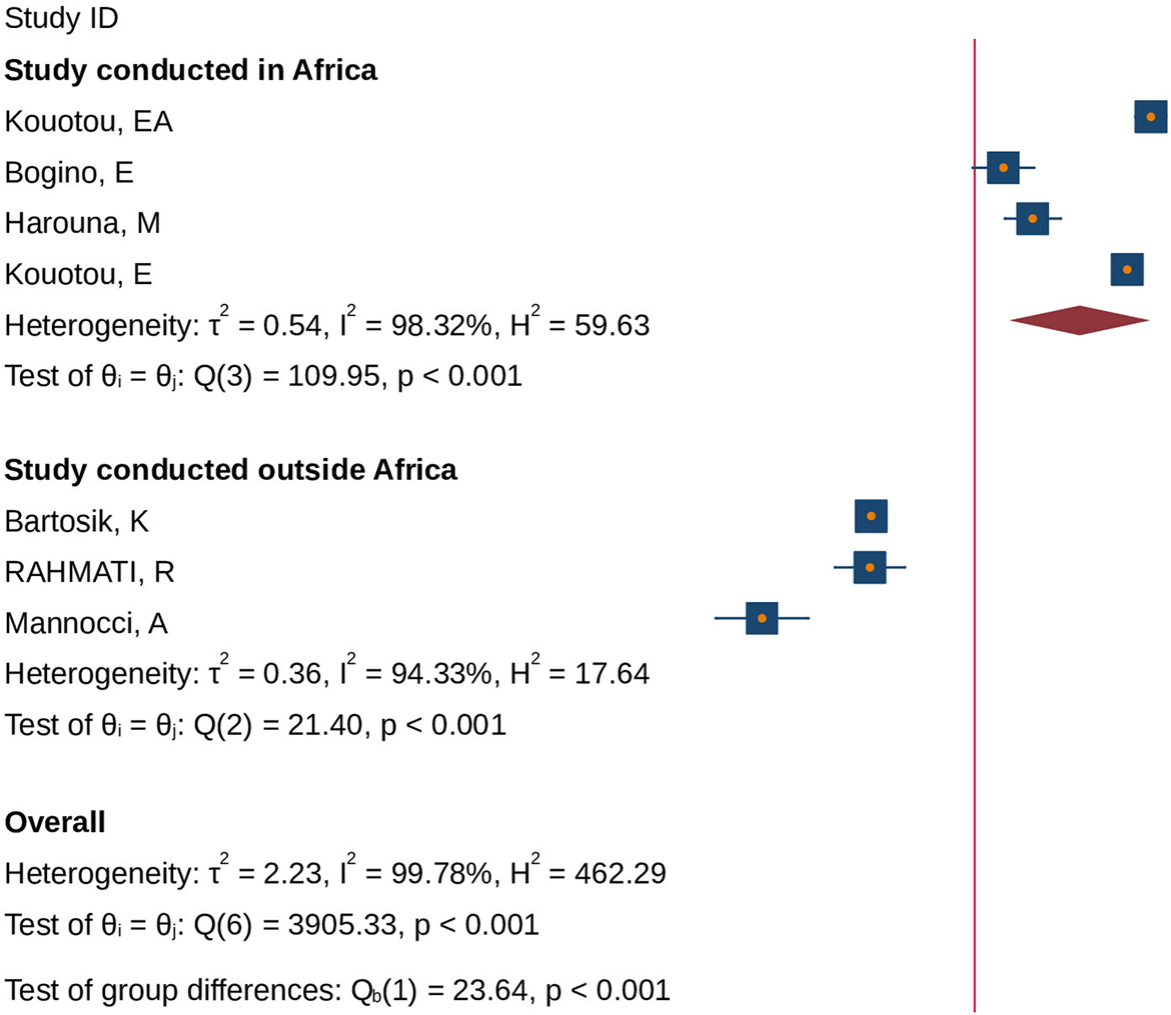
The pooled global prevalence of human scabies among prisoners based on the study area, 2024

By considering the heterogeneity of included studies in our meta-analysis, we conducted subgroup group analysis based on the study setting. According to subgroup analysis, the overall prevalence of scabies among African prisoners was 19.55% (95% CI; 9.44–40.45), while the prevalence among prisoners outside of Africa was 1.57% (95% CI; 0.77–3.19) (Fig. 3).

### Predictors of scabies among prisoners

A study conducted in Ethiopia [46] found that the length of time spent in prison is significantly associated with the occurrence of scabies. Prisoners who had been in prison for less than two months were 4.53 times [AOR: 4.53 (95% CI 1.51, 13.54)] more likely to develop scabies compared to those who had been there longer. Similar findings were observed in studies conducted in Iran [47] and Niger [48]. Based on a study conducted in Italy [35] also showed that there was a statistically significant association between the length of time spent in prison and the occurrence of human scabies.

This systematic review also found that hygienic practices were significantly associated with the occurrence of scabies among prisoners, based on included studies [44, 46, 48]. Based on a study conducted in Ethiopia [46], prisoners who did not use soap during hand washing had 5.53 times [AOR = 5.53; 95% CI: 1.45–21.17] higher odds of exhibiting scabies. A study conducted in Cameroon [44] also found that not bathing daily was associated with 1.23 times [AOR = 1.23; 95% CI: 2.10, 60.06] higher likelihood of developing scabies, while not doing laundry weekly was associated with 16.27 times [AOR = 16.27; 95% CI: 4.21, 62.84] higher likelihood. A study conducted in Niger [48] also found there was a significant association between hygiene factors, like toilet usage and soap usage, and the presence of scabies.

Sharing clothes or bedding with other inmates was also found to be significantly associated with scabies occurrence in studies conducted in Cameroon [36] and Ethiopia [46]. In Cameroon [36], inmates who shared clothes or bedding with other prisoners were 2.71 times [AOR = 2.71; 95% CI: 1.81, 4.06] more likely to develop scabies compared to those who did not share these items. Moreover, a study in Ethiopia [46] found that prisoners who did share clothes were 3.81 times [AOR = 3.81; 95% CI (1.09, 13.29)] more likely to develop scabies compared to those who did not share clothes.

Based on included studies [36, 47], overcrowding in prisons was found to be significantly associated with scabies, with a higher number of detainees per cell increasing the likelihood of scabies. A study in Cameroon [36] revealed that prisoners who had more than 10 people in their cell were 1.89 times [AOR = 1.89; 95% CI: 1.25–2.84] more likely to have scabies compared to prisoners with fewer people in their cell. A study conducted in Iran [47] revealed a statistically significant association between the number of roommates and the likelihood of experiencing scabies.

### Publication bias assessment

**Fig. 4:**
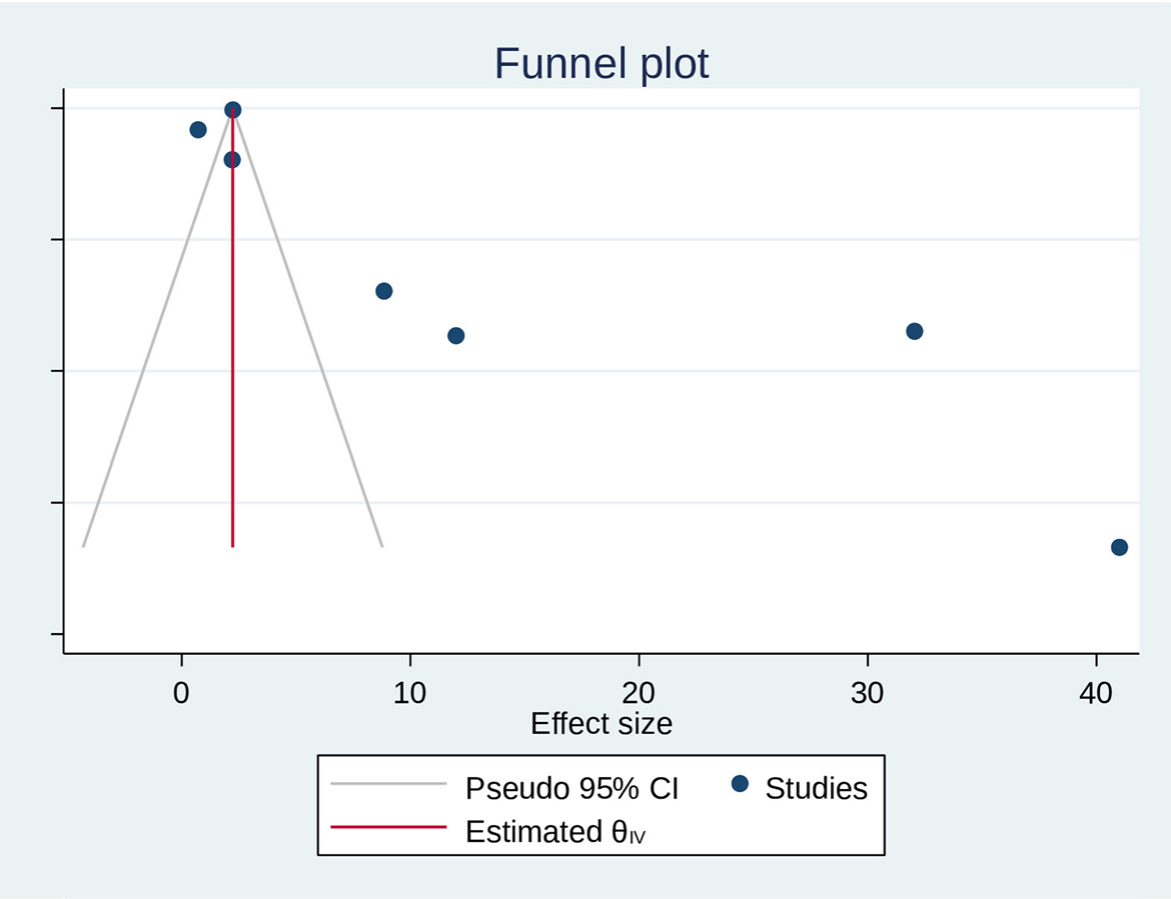
Funnel plot on the pooleds prevalence of human scabies among prisoners, 2024

**Fig. 5:**
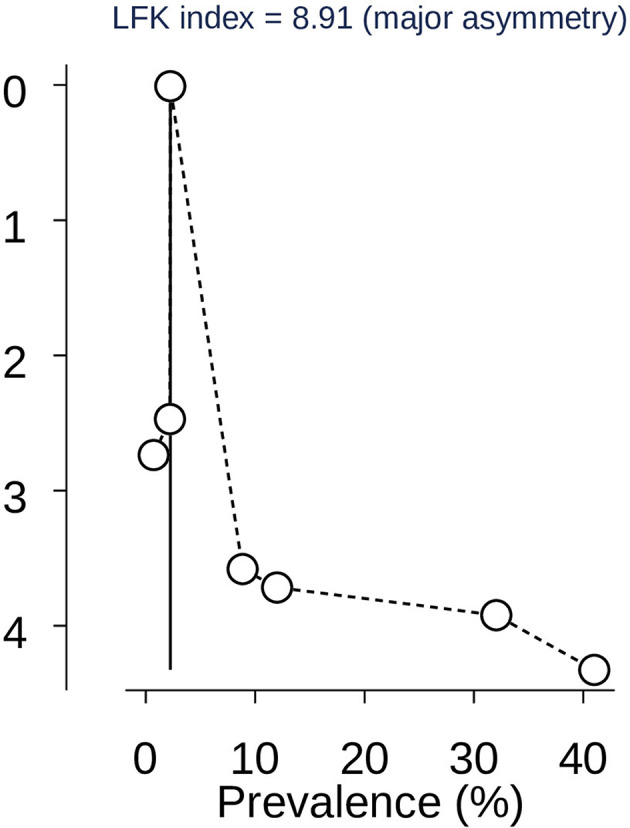
Dol plot analysis and LFK index of publication bias assessment for the pooled global prevalence of scabies among prisoners in 2024

The researchers checked for publication bias by visually inspecting a funnel plot, as well as using statistical tests. The funnel plot showed that the included studies were distributed asymmetrically. However, both Begg’s and Egger’s tests indicated the absence of publication bias in the global pooled prevalence of human scabies among prisoners. The tests showed no statistical evidence of publication bias with a p-value greater than 0.05 (P value; Eggers test = 0.21, Beggs test = 0.23), and the funnel plot was asymmetrical (Fig. 4). Funnel plots, Egger’s, and Begg’s tests are ineffective in detecting publication bias in a meta-analysis of proportions. Instead, Doi plots and the LFK index are suggested as better alternatives [52]. Accordingly, the Doi plot revealed a major asymmetry (LFK index = 8.92), indicating the presence of publication bias in the data (Fig. 5). Thus, it is crucial to be careful when interpreting the results of this systematic review and meta-analysis to account for the possibility of publication bias.

### Sensitivity analysis

**Fig. 6:**
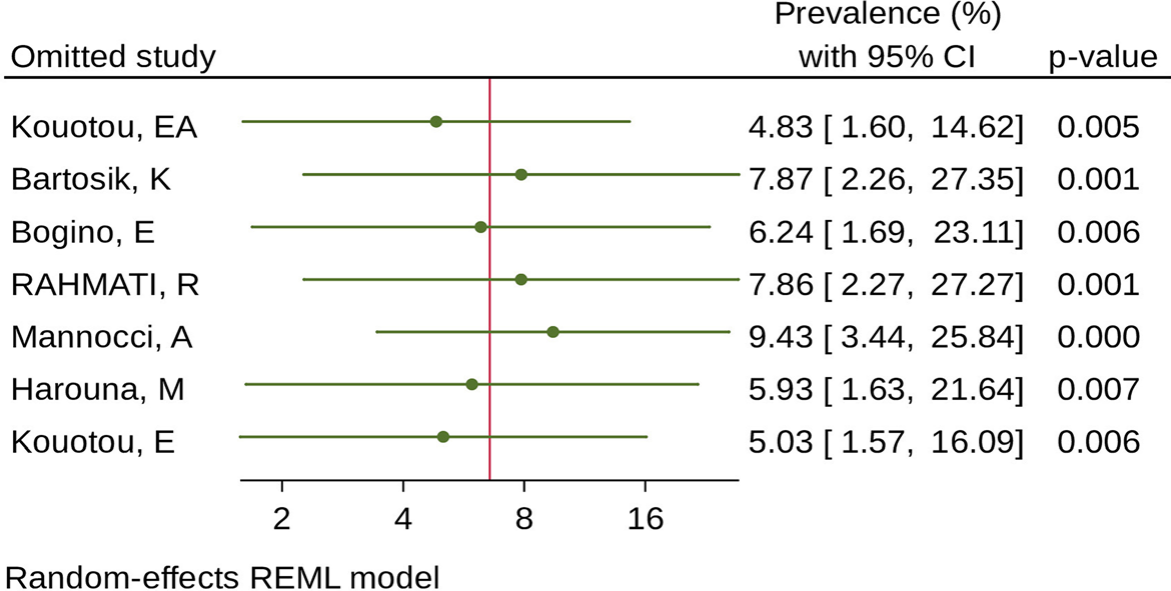
Meta leaves one out sensitivity on the pooled global prevalence of human scabies among prisoners, 2024

The result of sensitivity analyses revealed that none of the studies included influenced the overall estimate (Fig. 6).

## Discussion

Although the WHO guideline recommends people who are in prison have the same right to health care as everyone else, the majority of prisoners in several countries have challenges easily accessing health services as compared to the community [53]. Many people come into prison with medical and mental health problems that, if left untreated, can spread within the prison and be carried back into the community upon their release [54].

This systematic review and meta-analysis revealed that the pooled global prevalence of human scabies among prisoners was found to be 6.57% (95% CI; 2.16–19.94). These findings might be due to the persistent overcrowding of prisons found all around the globe, leading to an increase in the number of prisoners per cell. Inmates being in physical contact with each other in cells (e.g., at meals, on walks, and at work in the prison) and with prison Staff, medical personnel, or visitors [55].This finding align with another systematic review and meta-analysis that looked at scabies prevalence in the general population, which found that 14% of people worldwide have scabies [34]. Based on subgroup analysis, the pooled prevalence of scabies among African prisoners was found to be 19.55% (95% CI; 9.44–40.45), while among prisoners outside of Africa, it was 1.57% (95% CI; 0.77–3.19). A subgroup analysis found a higher prevalence of scabies in African prisons compared to countries outside of Africa, likely due to poor hygiene and conditions in African detention centers [56]. This finding was also supported by previous global systematic reviews that found that the prevalence of scabies varied widely, but was generally higher than 10% in all regions except Europe and the Middle East [27, 33]. This variation in scabies prevalence could be attributed to factors such as the sociodemographic and behavioural traits of the individuals being studied, as well as the specific settings in which the research was conducted. Due to a limited number of studies from tropical countries being included in our review, and considering that scabies infections are more prevalent in tropical and impoverished regions with limited access to water, where overcrowding and high temperatures promote the spread of the scabies mite [7, 27], the actual prevalence of scabies among prisoners may be higher than reported.

This systematic review examined various factors associated with the likelihood of scabies among prisoners. The analysis found that the amount of time spent in prison, hygiene practices, sharing of clothes or bedding, and overcrowding in prison cells were all significant predictors of scabies among prisoners. Accordingly, prisoners who had been in prison for less than two months were five times [AOR: 4.53 (95% CI 1.51, 13.54)] more likely to develop scabies compared to those who had been there longer. The possible justification for this could be that prisoners who are relatively new to the prison environment may be more prone to contracting scabies and prisoners who have been incarcerated for a longer period may have built up immunity to the infestation.

Prisoners who did not practice good hygiene, such as not using soap when washing their hands or not bathing daily, were more likely to get scabies. Similar findings were also found in Nigeria [57] which showed the frequency of baths and frequency of soap usage were significantly associated with the presence or absence of scabies. If people in prisons don’t practice good hygiene, like bathing regularly, using soap while washing hands, and cleaning their clothes, the chances of scabies spreading can be increased. This is because prisons have crowded living conditions, which makes it easier for scabies mites to pass from one person to another through close contact.

Sharing clothes or beddings with other inmates was also found to be significantly associated with scabies occurrence. This can significantly increase the likelihood of scabies occurrence primarily transmitted through direct skin-to-skin contact with an infected individual. Sharing clothes or beddings increases the opportunities for this type of contact, allowing scabies mites to transfer from one person to another. The act of inmates sharing clothes or beddings can lead to the easy spread of scabies in prison. These findings were supported by a study conducted in Ethiopia [58]. A previous review conducted globally also revealed that scabies can be transmitted through beddings, towels, and clothing [13].

The review concluded that prisoners in overcrowded cells are at a higher risk of contracting scabies compared to those in less crowded cells. The close physical proximity of inmates in crowded spaces facilitates the spread of scabies mites through physical contact, making overcrowded environments more conducive to the transmission of the skin condition. This finding is also supported by previous studies conducted in the Fuji trial [59]. It was also found that scabies was significantly associated with poverty and overcrowding [7, 8].

### Strengths and limitations of the study

This study was the first to investigate the worldwide prevalence of scabies among prisoners and its contributing factors, but this review only looked at those articles published in the English language. Besides, the results obtained from our systematic review and meta-analysis may not be fully representative on a global scale, as the included studies were limited to only six countries.

## Conclusion and recommendations

The overall prevalence of scabies among prisoners was found to be high globally. Factors such as the length of time spent in prison, sharing of clothing or beddings, personal hygiene habits, and overcrowding were identified as associated factors for scabies occurrence among prisoners. Efforts to reduce this risk involve encouraging good personal hygiene habits, ensuring inmates have access to clean clothes and beddings, establishing effective laundry procedures, and educating people about the significance of not sharing personal belongings. Taking measures to alleviate overcrowding is also crucial for reducing the prevalence and transmission of scabies among incarcerated individuals. The WHO and the International Committee of the Red Cross focus on healthcare in prisons and should prioritize implementing effective prevention and control measures to control and eliminate scabies.

### Electronic supplementary material

Below is the link to the electronic supplementary material.

Supplementary Material 1

## Data Availability

Data is provided within the manuscript or supplementary information files.

## Abbreviations

AOR: Adjusted odds ratio
CI: Confidence interval
PRISMA: Preferred reporting items for systematic reviews and meta-analysis
WHO: World health organization
